# A Model for Comprehensive Material Degradation and Strength Characteristics Assessment of Historic Masonry Structures

**DOI:** 10.3390/ma19112250

**Published:** 2026-05-26

**Authors:** Czesław Miedziałowski, Romuald Szeląg, Adam Walendziuk

**Affiliations:** Department of Building Structures and Structural Mechanics, Faculty of Civil Engineering and Environmental Sciences, Bialystok University of Technology, Wiejska 45E, 15-351 Białystok, Poland; c.miedzialowski@pb.edu.pl (C.M.); r.szelag@pb.edu.pl (R.S.)

**Keywords:** historic masonry buildings, assessment of material strength and structural safety, modelling of damaged heterogeneous materials

## Abstract

Historic buildings are susceptible to damage resulting from material degradation, deterioration of structural properties, changes in loads, overloading, and extraordinary events, which manifest as defects and flaws. These structures are also modernised, rebuilt, and adapted to new functions. In some cases, a rapid assessment of the structural condition is necessary. In practice, it is essential to maintain the safety of structures and their components, which is achieved by conducting an inventory and assessing the technical condition of structural elements and built-in materials. A numerical model was developed using macro-elements, and the practical applications of this model in strength analyses of degraded materials and sections of masonry structures are presented herein. The proposed approach was generalised and extended to include a classification of damaged masonry structures, which can be helpful and applied in indexing and searching databases of building structures, as well as in AI-assisted automatic pattern recognition. This resulted in a coherent and comprehensive approach to assessing the technical and structural condition of historic masonry buildings in use, as well as those undergoing repair, reconstruction, and modernisation.

## 1. Introduction

Small-scale elements such as bricks and stones have been used by builders for millennia to erect structures. Today, structures built in past centuries are still in use, retaining their original form. Often, architectural objects are treated in modern times as monuments of great historical value and are subject to special protection. The long-term use of masonry structures contributes to damage as a result of the natural degradation of materials and a reduction in their strength parameters, but also due to changing loads over different periods of use. It is often necessary to carry out ongoing maintenance, repairs and strengthening of such structures.

In this process, in addition to testing the strength of materials incorporated into the structure, it is important to carry out analyses to verify the strength and safety of historic masonry structures and their fragments. The parameters and factors affecting the strength of masonry structures, structural systems and material solutions used in masonry construction are described in studies [1,2,3,4,5], among others.

Historic buildings are also modernised and adapted to new functions, and their great historical value necessitates the preservation of their original features and the requirements defined by a heritage conservator. These processes involve inventorying, monitoring, and non-destructive, semi-destructive, and destructive testing of structural elements [6,7,8,9,10]. In this context, new technologies such as historic building information modelling and structural health monitoring have proven useful for obtaining information about the material and structural condition of buildings and their components [11,12,13,14]. These technologies play an important role in assessing the structural condition, identifying existing damage [15], and determining appropriate remedial measures and repair methods [16,17,18,19]. In the future, the data collected and analysed will form a resource of technical knowledge regarding the repair methods employed, thereby demonstrating the effectiveness of the solutions adopted and enabling the elimination of methods that have proved ineffective. This is important because most existing historic buildings lack documentation that would allow us to identify past works and structural alterations and determine their impact on the current technical condition and structural integrity of the building. In engineering practice, there are also instances where a rapid and comprehensive assessment of the technical condition of masonry structures and their components is required. This may occur during renovation and modernisation work on structural elements.

This article expands on the content of [20] in the context of the issues discussed above, including the application of the developed numerical model to assess the material properties and strength of historic structures and their components. The aforementioned article addressed issues related to the modelling of masonry structures, as well as details of the strength model developed using macro-elements. Without repeating the information, the authors will merely mention that the strategies used in the modelling of masonry structures aimed at determining the stress–strength state, as well as issues of numerical modelling, can be found, amongst others, in works [21,22,23]. Further details regarding the proposed method of modelling masonry structures, which will be presented to the extent necessary later in this article, can be found in [20]. The article also includes examples of numerical experiments confirming that the proposed method of modelling masonry structures yields good agreement between calculated and experimental results.

**Figure 1:**
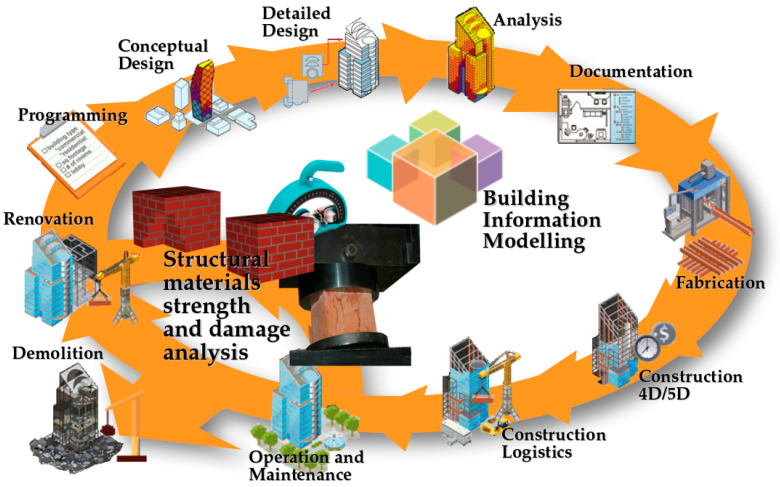
The issues discussed in the context of the general BIM relationship scheme.

The basis for developing a numerical model is the inventory and spatial and material identification of the historic building, which, in addition to traditional macroscopic measurements, utilises total station surveying, photogrammetry and 3D terrestrial laser scanning [6]. The preparation of documentation using BIM technology creates a database on the historic building, providing consistent documentation that remains accessible throughout its subsequent years of use. The digital model created also enables the development of a structural analysis model of the existing building and the incorporation of any new functional, design and revitalisation assumptions into it. Numerical analysis, combined with stress calculations for the structural elements, aims to avoid the need for demolition and to develop renovation solutions. To illustrate the issue in question, a BIM diagram supplemented with the scope of strength analyses of structural elements in damaged condition was used, as shown in Figure 1.

## 2. Assessment of the Technical Condition and Strength of Masonry

### 2.1. Technical Aspects

The materials used in construction are subject to progressive degradation, resulting in a deterioration of their original properties and parameters over time. The causes of this are physical, chemical, and biological in nature. In addition to mechanical damage to walls, during the operation of a building, the loads acting on it change; some parts of the structure are overloaded, while others are relieved, resulting in a redistribution of stresses in the materials built into the structure. This is important in reconstruction processes when it is necessary to locally replace damaged elements with new ones.

In many regions of the world, an important factor in destruction is the dampness of walls and the processes of freezing and thawing of ceramic materials and mortar. Among the biological causes, factors related to mycology and lichenology, which have a long-term impact, are indicated.

**Figure 2:**
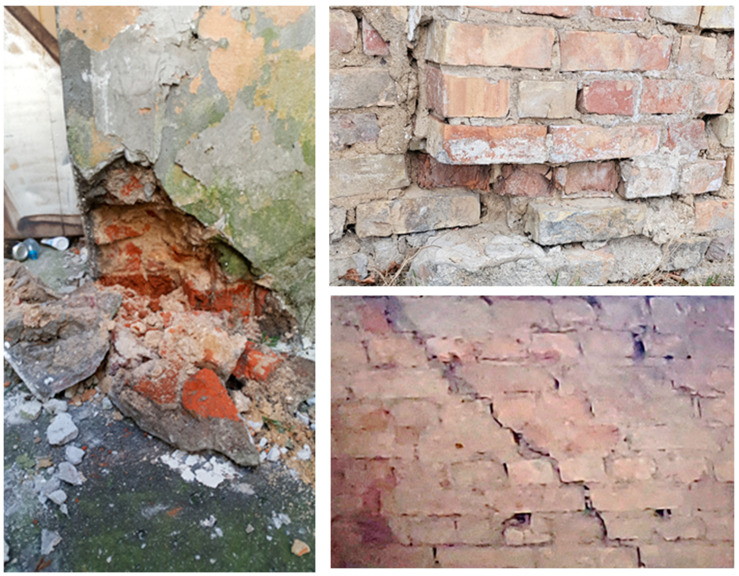
Various technical conditions of masonry fragments caused by degradation processes.

Most often, the degradation of the components of masonry structures is caused by the simultaneous action of many of the above-mentioned factors, resulting in mass losses in the wall structures. The consequence of this process is fragments of the structure with reduced strength and poor technical condition, as shown in Figure 2. The degradation process is inevitable and difficult to stop, occurring in historic buildings over decades. A good illustration of the problem can be found in layered walls (including degraded ones), in the description of which segments in various technical conditions should be distinguished. The segmentation process may result from structural damage to the bricks, which may be accompanied by their disintegration from the structure, or a local cracking process separating continuous structures into local areas due to the occurrence of permanent cracks.

In order to standardise the strength analysis procedure, walls with a regular arrangement of masonry units are considered. The proposed method of description consists in separating segments of a certain size, referred to as macro-elements, in the model of the analysed structure. The macro-elements assume the actual state of degradation, taking into account the variability of physical parameters in the subspaces of the structure and damaged fragments, introducing limits on the variability of material parameters and damage. In this way, it is possible to optimally model the properties of construction materials in a 3D diagram, e.g., using the finite element method.

### 2.2. Photogrammetric Technique

In the process of locating damage to masonry structures, the representative process of determining areas with different strengths of bricks and mortar, and subsequent classification into the appropriate calculation model, Structure-from-Motion (SfM) photogrammetry has proved to be effective [24,25,26,27,28]. This technology is based on taking a series of photographs at different angles in relation to the structures under investigation in order to obtain a final three-dimensional image that enables the implementation of automation procedures at a later stage. The technology works well for creating images of damaged wall areas, which, due to the degree of degradation, may pose a safety hazard in conditions of direct access [29,30,31,32], but it is also recommended for use due to its low cost [33]. The interpretation of the point cloud obtained from the method can be used to divide it into separate regions, which allows for independent analysis of each segment [34]. This approach has been effectively used to prepare data for the calculation of masonry structures in computational areas of walls using the finite element method [35].

**Figure 3:**
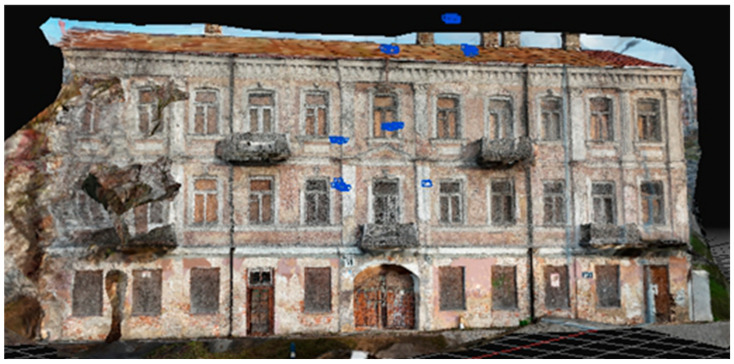
Masonry structures of the front wall used in material strength analysis obtained using SfM technology. The analysis was performed using 3DF Zephyr Free 8.031 software.

**Figure 4:**
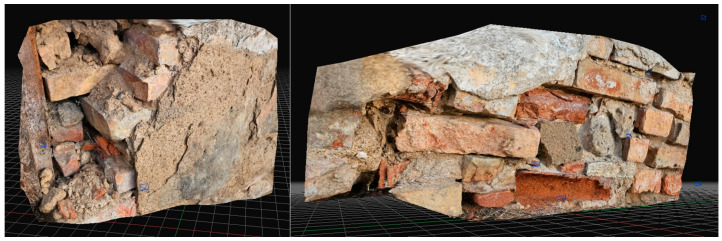
Isolated areas with reduced strength parameters in masonry structures subjected to detailed strength tests.

In the process of preparing to identify locally different material parameters, it is effective to perform imaging of the analysed structures using SfM technology. This provides a comprehensive image of the entire object and enables material testing in all selected locations without the risk of weak areas being overlooked (Figure 3). Analyses conducted in this manner precede in-situ material tests and are performed after preliminary processing of the results and the creation of a model of structures with similar technical parameters, indicating areas of damage, weld defects, cracks or spalling of the substance, and preliminary classification of wall damage. The correct preparation and selection of research sites is aimed at correctly determining the material parameters of the tested areas, which will later be interpreted as zones with characteristic representative properties. The comprehensive scope of analyses conducted using photogrammetry enables the location of damaged areas for further testing. In this regard, the work carried out aims to determine the type of damage to the walls, the strength parameters of the structures, and, due to the presence of brick and mortar losses, individualised calculation models saturated with strength parameters. An example of identifying areas with different characteristics is shown in Figure 4, which was created using photogrammetry.

When SfM procedures are applied, attention is drawn to practical challenges that may arise during the mapping of masonry walls, particularly in homogeneous areas such as window panes. This is due to the difficulty of tracking unique feature points. Furthermore, extended image acquisition under variable lighting conditions, where image sequences may include shadowed wall sections, can cause matching algorithms to incorrectly overlap fragments of the model.

The loss of bricks and mortar, as well as damage to surface layers, indicates the need to develop computational models which, after material testing, will allow an assessment of the impact of masonry discontinuities on local stress increases and damage propagation.

### 2.3. Material Testing

**Figure 5:**
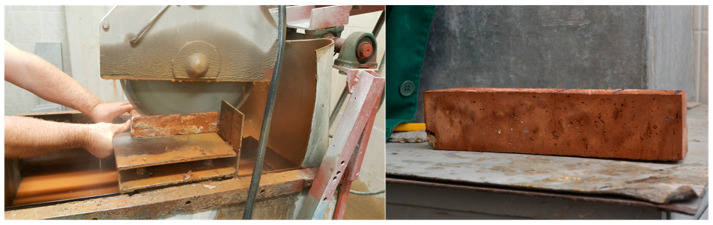
Test samples were prepared by cutting cubes from the brick material and then seasoning them in accordance with the provisions of standard [36].

Brick strength tests were carried out in accordance with the standard [36]. The preparation of elements for testing consists in preparing samples from the tested brick from areas that do not have surface damage and then seasoning them to an air-dry state for a period of 14 days at a temperature of ≥15 °C with a relative humidity of ≤65%. The initial stage of preparing samples for testing is shown in Figure 5.

**Figure 6:**
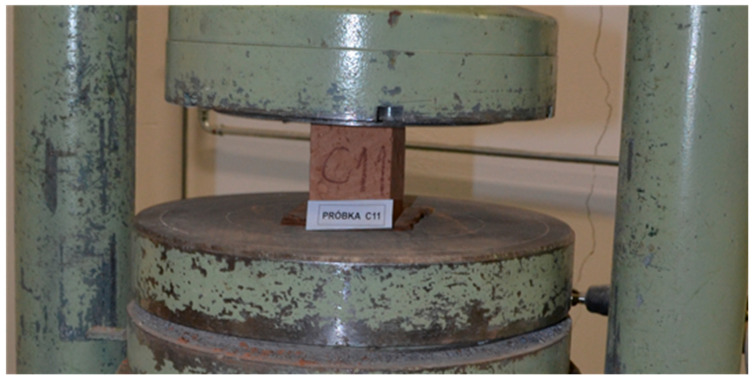
Strength tests were carried out in a strength testing machine, with the load increment set at a speed of 0.3 (N/mm^2^)/s.

The prepared samples were measured with an accuracy of 0.1 mm and placed in a strength press. The height of the samples was h = 59.0 mm, while the transverse dimension ranged from 50.0 to 50.5 mm. Up to 8 samples for strength testing could be prepared from each brick. The process of testing the strength of one of the samples is shown in Figure 6.

**Figure 7:**
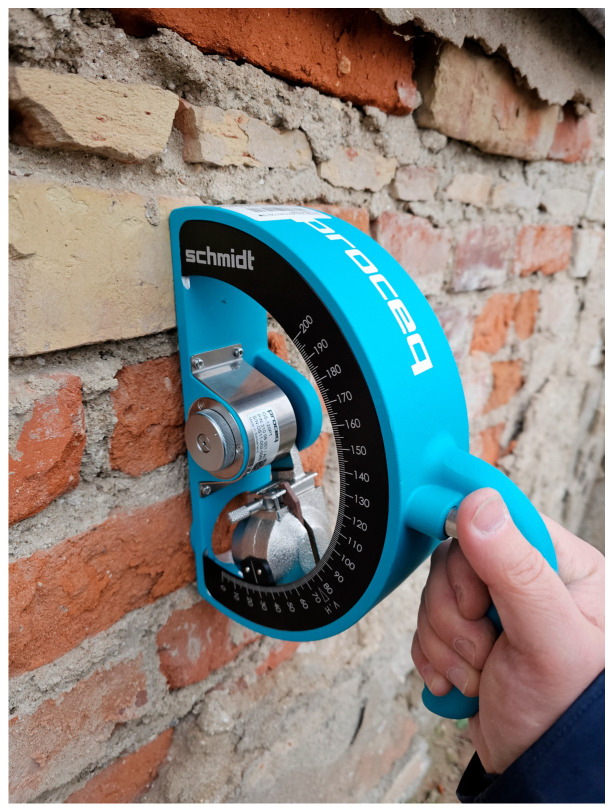
Non-destructive strength testing of low-strength bricks was carried out using a Schmidt hammer of type OS-120PT (Proceq, Schwerzenbach, Switzerland). It allows the testing of materials with strength ranging from 1 to 5 MPa.

When the strength of a test sample is determined, the shape factor “d” is used to reduce different dimensions to a common comparative value. For the test samples, parameter d equal to 0.90 was used. In masonry structures where there was no surface damage, the normalised strength for samples taken from solid brick was within the range of $f_{b\;}=13.1÷15.8\;N/{mm}^{2}$, with an average value of $f_{b\;}=14.4\;N/{mm}^{2}$; this indicated a reduction in their strength and allowed them to be classified as class 10. Areas where low brick strength was identified were tested using the sclerometric method [37], with a Schmidt hammer of type OS-120PT (Figure 7), in order not to weaken them.

The performance of non-destructive testing requires the derivation of a correlation equation, the coefficients of which are obtained from destructive testing. Alternatively, it is possible to use a standard curve in the form of an equation [38]:

(1) $$f_{b}(i)=1.05\cdot (0.0015R_{i}^{2}+0.0615R_{i}-0.3585)$$

where $f_{b}(i)$ is the specified strength in $\left[N/{mm}^{2}\right]$, and $R_{i}$ is the average measured Schmidt hammer rebound at the measurement point. Measurements carried out on bricks with similar characteristics of damage to the outer layers enabled the test results to be compiled and the strength of the bricks to be determined (Table 1).

The results of parameter testing were prepared in accordance with the ITB instructions [40], specifying:

the average number of rebounds

$$\bar{R_{i}}=28.78$$

the standard deviation of the rebound

$$s\left(R\right)=1.523$$

the rebound number variability coefficient

$$v\left(R\right)=5.292\%.$$

the average strength of bricks using the standard function

$$f_{b}(i)=2.79\;N/{mm}^{2}.$$

the standard deviation of strength

$$S_{R}=0.236\;N/{mm}^{2},$$

the minimum strength

$$f_{bmin}(i)=2.40\;N/{mm}^{2}.$$

the coefficient of variation

$$v_{R}=8.469\%,$$

and the area homogeneity coefficient

$$k_{R}=0.861.$$

The non-destructive testing report leads to the determination of the $f_{bmin}(i)$ strength, which is the basis for the classification of bricks and is a parameter used in the process of creating a calculation model. During the research, it was noticed that in places where the joints did not provide adequate coverage of the bricks, due to their destruction, the bricks were displaced by the impact of the Schmidt hammer. Such places do not ensure proper interaction of materials in wall structures. For bricks with low strength, the sclerometric method using the OS-120PT hammer can be useful for determining their compressive strength. The strength of mortar in masonry can be tested in a similar way to that presented, using a Schmidt hammer similar to the one presented but of type OS-120PM, which has a different tip. The determined strength of the lime mortar was M 0.5 in areas where it retained its technical parameters, and in weakened areas, its strength was close to 0.1 MPa.

### 2.4. Method of Analysis

**Figure 8:**
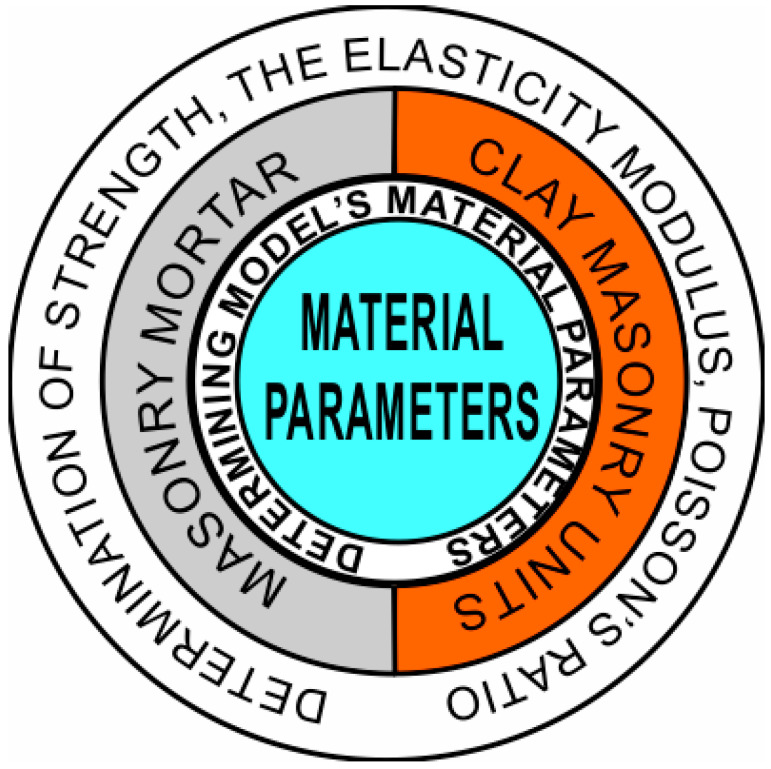
The identification of mortar and brick strength parameters used in the process of creating computational models of masonry structures.

In masonry structures, as a result of long-term use accompanied by processes that deteriorate their technical condition, there are fragments that are degraded and damaged to varying degrees. Degradation is understood as a local change in the stiffness of the material, manifested by a loss of mass, cracking, surface flaking and, consequently, changes in the strength parameters of the masonry. Damage causing a break in the continuity of the masonry also excludes the damaged areas or volumes from static operation. It is important to determine the current strength parameters of the tested structures in relation to bricks and mortar. In the process of preparing the calculation model, it is essential to determine the parameters described in Figure 8.

**Figure 9:**
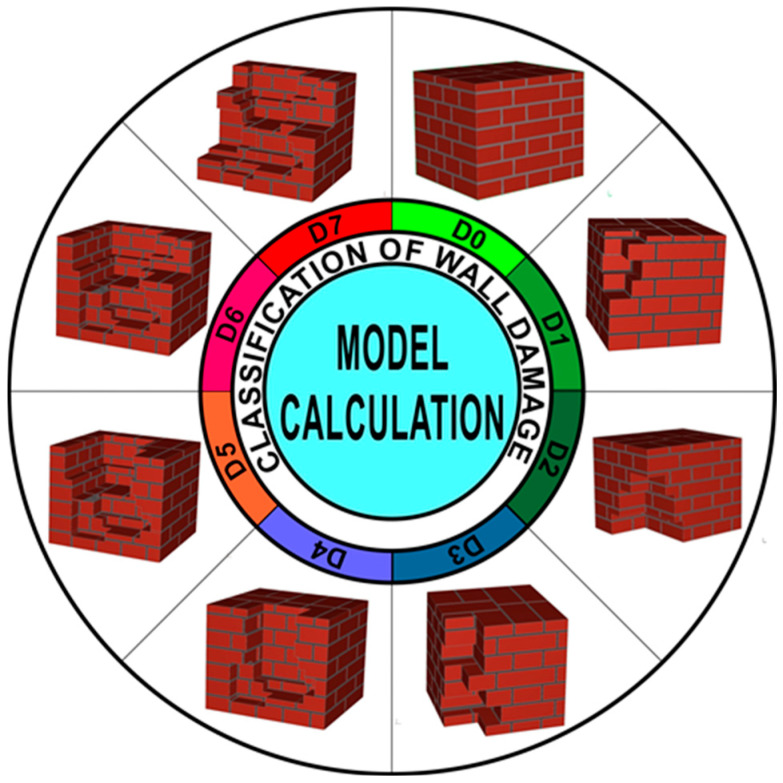
Classification of damaged masonry structures in 20-node macro-elements.

The next stage is the description of degradation and damage analysed on two scales: on the structural scale, an equivalent homogeneous medium is considered, and on the mesoscale, the complex heterogeneous mesostructure of the wall is taken into account. Reductions in the parameters of the wall mesostructure are systematised, in relation to the degree of damage occurring, with the introduction of an eight-level classification of damage. The D0 ÷ D7 classification proposed in this study indicates the identified level of damage, from D0, corresponding to no damage to masonry structures, up to level D7, which is equated with damage at a level close to 88%. At each level of the scale, the damage increases by 12.5%, which limits the number of macro-elements developed to a closed database of elements. The proposed classification was developed for engineering practice purposes and requires further calibration and validation through research conducted on real-scale structural members. The classification concept prepared in this way is explained in Figure 9, which shows representative macro-elements used in numerical analyses.

**Figure 10:**
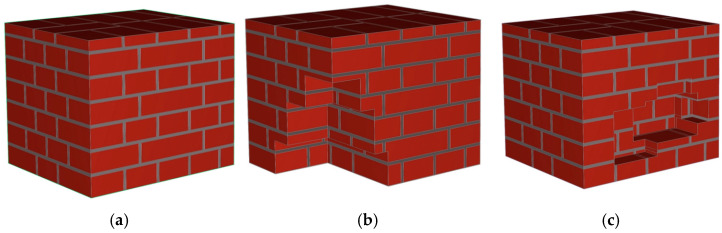
Examples of internal structures of macro-elements: (**a**) undamaged D0; (**b**,**c**) with various forms of degradation or damage, corresponding to class D2.

**Figure 11:**
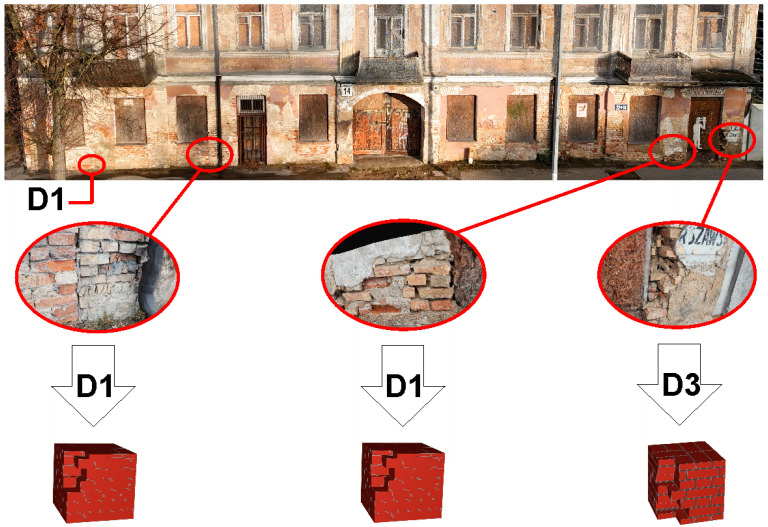
Classification of wall damage to the developed scope; elements of classes D1 and D3 are present.

The proposed method of description involves separating segments of a given size, referred to as macro-elements, within the model of the analysed structure. The macro-elements reflect the actual state of degradation, taking into account the variability of physical characteristics in the subspaces of the structure and damaged fragments by introducing limits on the variability of material parameters and damage. Examples of internal structures of macro-elements are shown in Figure 10. This allows for optimal modelling of the properties of structural materials in a 3D diagram, e.g., using the finite element method. The use of macro-elements and stage analyses allows for avoiding computational tasks with a large number of unknowns. In the initial stage, it is important to identify the degree of damage and assign it to the appropriate class, as explained in Figure 11.

The location of damage to masonry structures should begin with areas that are particularly exposed to the effects of rainwater and areas of contact with the ground, where the effects of melting snow and water splashes from raindrops can accumulate [41]. If areas of masonry degradation are found, the remaining areas should be examined in detail in order to properly classify the actual masonry structures by assigning them appropriate macro-element models.

The next stage is to apply the developed stiffness matrices of macro-elements $K_{e}$ obtained by integration in subspaces according to the formula

(2) $$K_{e}=\int_{V_{1}}B^{T}E_{1}^{\left(t\right)}B\mathrm{dV}+...+\int_{V_{j}}B^{T}E_{j}^{\left(t\right)}BdV+\cdots +\int_{V_{n}}B^{T}E_{n}^{\left(t\right)}BdV$$

while the stiffness of the entire structure is described by the set of all macro-elements. The sub-elements of the integration are the material fragments of the actual features used in the masonry structure. In Equation (2), ***B*** is the deformation matrix, and the time-varying elasticity matrix of sub-area *j* (bricks, joints) is written in the form:

(3) $$E_{j}^{\left(t\right)}=E_{j}=\frac{E_{j}}{\left(1+\nu_{j})(1-2\nu_{j}\right)}\left[\begin{array}{cccccc} \left(1+\nu_{j}\right) & \nu_{j} & \nu_{j} & 0 & 0 & 0\\ \nu_{j} & \left(1+\nu_{j}\right) & \nu_{j} & 0 & 0 & 0\\ \nu_{j} & \nu_{j} & \left(1+\nu_{j}\right) & 0 & 0 & 0\\ 0 & 0 & 0 & \frac{\left(1+2\nu_{j}\right)}{2} & 0 & 0\\ 0 & 0 & 0 & 0 & \frac{\left(1+2\nu_{j}\right)}{2} & 0\\ 0 & 0 & 0 & 0 & 0 & \frac{\left(1+2\nu_{j}\right)}{2} \end{array}\right]$$

In the designation of the matrix $E_{j}$ the superscript (*t*) is used to emphasise the variability of the parameters over time. The elements of the matrix, which depend on the material constants of the Young’s modulus *E* and the Poisson’s ratio *ν* of the brick and mortar, are, in the general case, variable in the assumed sub-areas *j* of these materials during the long-term service life of the structure and should be determined in laboratory tests.

The assessment of the safety condition of the structure is proposed to be carried out according to a stepwise algorithm that uses the formulation of the equilibrium equations in the following matrix form:

(4) $$K_{i}^{\left(t\right)}q_{i}^{\left(t\right)}=\;Q_{i}^{\left(t\right)}$$

where $K_{i}^{\left(t\right)}$ is the stiffness matrix of the modelled medium, $Q_{i}^{\left(t\right)}$ is the load vector, and $q_{i}^{\left(t\right)}$ is the vector of unknown displacements of the model nodes at step *i* of the iteration. The calculated displacement values are used to determine the deformations and stresses in material structures. The stress values, in turn, are the basis for verifying the strength of bricks and mortar. Failure criteria are used to assess the strength of bricks and mortar in macro-elements. The authors propose the use of the Maxwell–Mohr criterion and, in the case of materials under tension, the Rankine criterion. The formal notation of the failure criteria used is as follows:

(5) $$\frac{1}{2\cos\varphi }\left(\sigma_{i}-\sigma_{j}\right)+\frac{1}{2}\left(\sigma_{i}+\sigma_{j}\right)\tan\varphi <f_{s},\;\;\;\;\;\;\;i,j=1,2,3$$

**Figure 12:**
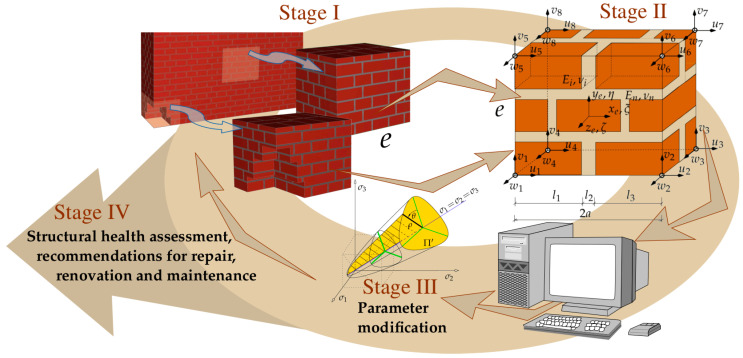
Stages of the calculation process for assessing the current technical condition of a masonry structure.

$\sigma_{i},\sigma_{j}$ are principal stresses, φ is the angle of internal friction and $f_{s}$ is the shear strength. At the joints between materials (bricks and mortar), stress and strength are checked using the following criterion:

(6) $$\left|c-\sigma_{n}\tan\varphi_{m}\right|<f_{sm}$$

where $\varphi_{m},f_{sm}$ are the mortar parameters, $\sigma_{n}$ is normal stress at the interface and c is the cohesion. The scheme for conducting the calculations is divided into stages illustrated in Figure 12.

The procedure used in the calculations comprises four stages of developing a structural model and assessing its technical condition. Stage I involves analysing large surfaces or volumes using photogrammetry and constructing macro-elements. The next stage, Stage II, involves analysing the state of displacement, stress and strain inside the macro-elements. Stage III involves analysing the results obtained and, if necessary, modifying the model parameters and repeating the calculations. The final stage IV involves proposals and recommendations for repairs and maintenance of the structure.

The stiffness matrices used in the numerical analysis of the brick and mortar materials, obtained from the strength parameters tested in the laboratory and tests using the OS-120 Schmidt hammer, are as follows:

(7) $$E_{1}^{\left(brick\right)}=\left[\begin{array}{cccccc} 4119 & 516 & 516 & 0 & 0 & 0\\ 516 & 4119 & 516 & 0 & 0 & 0\\ 516 & 516 & 4119 & 0 & 0 & 0\\ 0 & 0 & 0 & 2060 & 0 & 0\\ 0 & 0 & 0 & 0 & 2060 & 0\\ 0 & 0 & 0 & 0 & 0 & 2060 \end{array}\right],$$

(8) $$E_{2}^{\left(mortar\right)}=\left[\begin{array}{cccccc} 172 & 21 & 21 & 0 & 0 & 0\\ 21 & 172 & 21 & 0 & 0 & 0\\ 21 & 21 & 172 & 0 & 0 & 0\\ 0 & 0 & 0 & 86 & 0 & 0\\ 0 & 0 & 0 & 0 & 86 & 0\\ 0 & 0 & 0 & 0 & 0 & 86 \end{array}\right],$$

(9) $$E_{3}^{\left(brick\right)}=\left[\begin{array}{cccccc} 17164 & 2149 & 2149 & 0 & 0 & 0\\ 2149 & 17164 & 2149 & 0 & 0 & 0\\ 2149 & 2149 & 17164 & 0 & 0 & 0\\ 0 & 0 & 0 & 8582 & 0 & 0\\ 0 & 0 & 0 & 0 & 8582 & 0\\ 0 & 0 & 0 & 0 & 0 & 8582 \end{array}\right],$$

(10) $$E_{4}^{\left(mortar\right)}=\left[\begin{array}{cccccc} 858 & 107 & 107 & 0 & 0 & 0\\ 107 & 858 & 107 & 0 & 0 & 0\\ 107 & 107 & 858 & 0 & 0 & 0\\ 0 & 0 & 0 & 429 & 0 & 0\\ 0 & 0 & 0 & 0 & 429 & 0\\ 0 & 0 & 0 & 0 & 0 & 429 \end{array}\right].$$

$E_{1},E_{2}$—damaged areas of the brickwork; $E_{3},E_{4}$—brickwork with correct technical parameters. Values are given in units [MPa].

## 3. Assessment of the Load-Bearing Capacity of Masonry Structures Using Developed Calculation Procedures

Using a database of macro-elements adapted to the damage found in masonry structures, analyses were carried out to verify the correctness of the proposed approach in relation to a section of masonry forming the external façade wall of a historic building. The database should take into account the current condition and information related to the history of the building structure. In addition to the significant advantages of BIM technology in this area, its integration with continuous monitoring provides measurable benefits related to the ongoing updating and expansion of the database. Furthermore, it enables continuous assessment of the building’s mechanical performance and safety.

**Figure 13:**
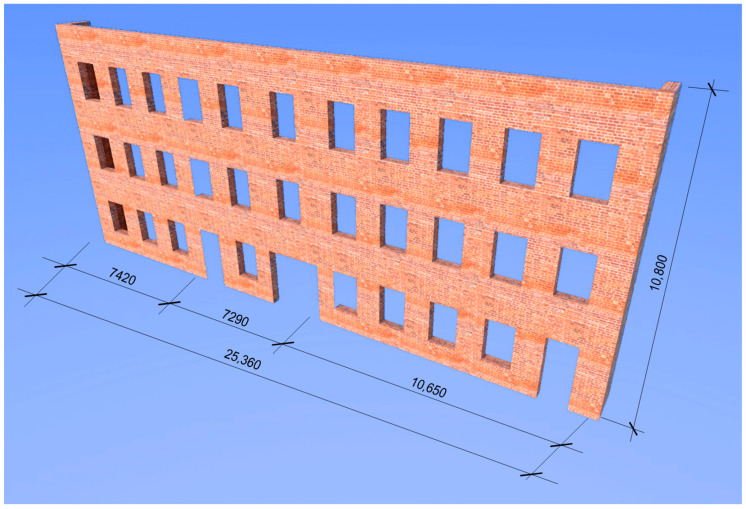
The structure of a brick wall analysed using the developed macro-elements.

The presented method of assessing the condition of a structure is illustrated by a strength analysis of a 7.42 m long and 51 cm thick section of a solid brick wall, which is part of the 25.36 m long front wall shown in Figure 13.

It is a structural element of a three-storey building without a basement, built of solid brick with lime mortar and cement–lime mortar, with a longitudinal wall structure. In order to assess its current strength, a numerical model of a fragment of the structure was developed in the form of a longitudinal wall with geometry corresponding to that of the entire structure. Macro-elements and the proposed method of modelling the structure were used in the construction of the numerical model.

**Figure 14:**
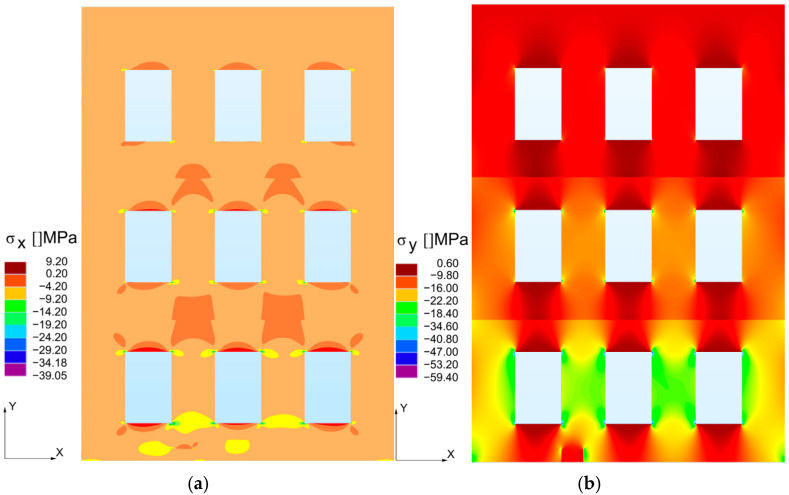
Computer model of a fragment of the structure and the results of stress analysis: σ_x_ (**a**) and σ_y_ (**b**) in the masonry structure.

As a result of a phased numerical analysis, results were obtained in the form of stress fields (Figure 14), which allow the current strength condition of the masonry structure to be assessed. It is also important to be able to compare the results of the analyses with the results of previous calculations. Over the years, the presented results will constitute a collection of archival analyses used to identify changes occurring in the wall structures and their impact on the safety of structural systems.

## 4. Conclusions

The continuous development of modern technologies is an undeniable fact. Such technologies are becoming increasingly noticeable and are actively entering many areas of the economy. They include both hardware technologies and software platforms.

In the field of civil engineering, BIM technologies and computational methods such as FEM have enormous potential for the analysis, maintenance and restoration of historic buildings. Thanks to their ability to create detailed digital models that take into account the heterogeneity of materials and their technical condition, and to perform advanced simulations, these techniques can help to better diagnose the static behaviour of historic structures and plan effective measures.

The integration of modern technologies enables the development of a database on the history of structural, repair and conservation changes to buildings, as well as monitoring of their current technical condition.

By combining the collected data on the technical condition [41] with numerical simulations, diagnostics can be expanded to include the technical condition of the structure, and the risk of further damage is minimised. However, the use of other modern technologies, apart from those mentioned in this article, to obtain information useful in assessing the technical condition and safety of structures remains an open question.

The use of photogrammetry significantly speeds up the process of developing macro-elements modelling masonry structures, allowing for the mapping of damage to individual bricks and areas with reduced technical parameters. The implementation of stiffness matrices with individually defined damage parameters enables the prediction of repair effectiveness. This is achieved by replacing damaged areas of the structure with finite macro-elements with actual geometric and strength parameters, thus identifying the local impact of the repairs in relation to the limit state of the entire masonry structure. This article proposes and describes a method for the comprehensive material and strength assessment of masonry structures with varying degrees of degradation. An attempt was made to formalise the technical assessment process for masonry structures, including technologies that are currently becoming standard in diagnostics of existing and newly designed buildings.

The application of a step-by-step diagnostic process based on numerical simulations of stress state changes in macro-elements, taking into account the state of damage, introduces new possibilities for determining the order of repairs in masonry structures characterised by significant damage. The intermediate stage, in which bricks are locally replaced with new ones during the repair process, is simulated using macro-elements with characteristics resulting from the remaining areas in relation to the technical condition of structures that have often been in use for hundreds of years. The development of a model using the proposed solutions speeds up the decision-making process, allows interference with historic structures to be kept to a minimum, and leaves masonry structures in good technical condition for future generations.

## Figures and Tables

**Table 1:** Results of sclerometric tests on wall bricks.

Object	Tenement house	Date of construction								19th century
		Expected grade of bricks								Less than 10 MPa
Element	Front wall. Damaged bricks	Schmidt OS-120PT pendulum								
		Calculation according to standard [39]								
No.	Angle α=0°	Rebound value R								Rebound value average $R_{i}$
		1	2	3	4	5	6	7	8	
1	V—vertical position of the hammer	25	26	25	27	26	25	24	25	25.4
2		29	28	29	30	31	30	31	30	29.8
3		30	31	32	30	29	30	31	30	30.4
4		27	26	30	29	30	29	30	29	28.8
5		29	30	30	28	30	31	30	31	29.9
6		30	30	29	28	30	31	29	30	29.6
7		28	27	26	28	28	29	30	27	27.9
8		27	28	27	28	29	27	29	28	27.9
9		27	28	29	29	27	28	29	28	28.1
10		30	29	31	30	30	30	30	31	30.1
										$\Sigma R_{i}=287.8$

## Data Availability

The original contributions presented in this study are included in the article. Further inquiries can be directed to the corresponding author.
